# Identification and predictability of soil quality indicators from conventional soil and vegetation classifications

**DOI:** 10.1371/journal.pone.0248665

**Published:** 2021-10-22

**Authors:** Paul Simfukwe, Paul W. Hill, Bridget A. Emmett, Davey L. Jones

**Affiliations:** 1 Department of Agricultural Biotechnology and Biosciences, School of Agriculture and Natural Resources, Mulungushi University, Kabwe, Central Province, Zambia; 2 School of the Environment, Natural Resources & Geography, Bangor University, Bangor, Gwynedd, United Kingdom; 3 Centre for Ecology and Hydrology, Environment Centre Wales, Bangor, Gwynedd, United Kingdom; ICAR-National Rice Research Institute, INDIA

## Abstract

The physical, chemical and biological attributes of a soil combined with abiotic factors (e.g. climate and topography) drive pedogenesis and some of these attributes have been used as proxies to soil quality. Thus, we investigated: (1) whether appropriate soil quality indicators (SQIs) could be identified in soils of Great Britain, (2) whether conventional soil classification or aggregate vegetation classes (AVCs) could predict SQIs and (3) to what extent do soil types and/ or AVCs act as major regulators of SQIs. Factor analysis was used to group 20 soil attributes into six SQI which were named as; soil organic matter (SOM), dissolved organic matter (DOM), soluble N, reduced N, microbial biomass, DOM humification (DOMH). SOM was identified as the most important SQI in the discrimination of both soil types and AVCs. Soil attributes constituting highly to the SOM factor were, microbial quotient and bulk density. The SOM indicator discriminated three soil type groupings and four aggregate vegetation class groupings. Among the soil types, only the peat soils were discriminated from other groups while among the AVCs only the heath and bog classes were isolated from others. However, the peat soil and heath and bog AVC were the only groups that were distinctly discriminated from other groups. All other groups heavily overlapped with one another, making it practically impossible to define reference values for each soil type or AVC. The two-way ANOVA showed that the AVCs were a better regulator of the SQIs than the soil types. We conclude that conventionally classified soil types cannot predict the SQIs defined from large areas with differing climatic and edaphic factors. Localised areas with similar climatic and topoedaphic factors may hold promise for the definition of SQI that may predict the soil types or AVCs.

## Introduction

The multiple roles and functions of soil have resulted in many broad definitions of soil quality. One of the most widely adopted definitions for soil quality (SQ) was proposed by a committee for the Soil Science Society of America as: “the capacity of soil to function, within natural or managed ecosystem boundaries, to sustain plant and animal productivity, maintain or enhance water and air quality, and support human health and habitation” [1]. Soil quality is evaluated in terms of measurable soil attributes that measure specific physical, chemical, and biological properties; also known as soil quality indicators (SQIs) [2–4]. Many of these properties are interrelated and the applicable SQIs are those that integrate and have the combined effect of several properties or processes that affect the capacity of a soil to perform a specified function [5–7]. The quality of any soil has two parts: (1) the natural or inherent quality which is based on the parent geological material and soil-state-factors (climate, topography, biota, and time) and is rather static, and (2) the dynamic soil quality which encompasses those soil properties that can change over relatively short time periods in response to human use and management [4, 6, 8, 9]. The inherent SQs, are used in taxonomic soil classification while the dynamic SQs are used to monitor temporal trends on the same soil. For that reason, measurement of key SQIs over time can be used to establish whether the quality of a soil under a given land use and management system is improving, declining or stable [2, 4, 10, 11]. The SQIs which respond over the medium term (sensitive over few years and decades) to land uses and management practices, may be the most useful for indicating such changes as opposed to those which change either very rapidly (e.g. seasonally) or very slowly (e.g. over centuries) [7, 12, 13].

Even though dynamic SQIs are not used in taxonomic classifications and soil surveys, they are greatly controlled by soil types [14]. Studies by Parkin [15], Buyer et al. [16], Girvan et al. [17] and Ulrich and Becker [18] have shown that soil type is a key factor determining many SQIs. Another key regulator of SQIs in the environment is the type and quantity of biota [19, 20]. The type and quantity of biota (vegetation cover) determines the kind and quantity of organic materials that are returned in the soils which in turn regulates many biophysical and chemical soil attributes. The biota type influences the spatial distribution of the organic materials in the soils. For instance over 12 Mg ha^-1^ dry matter may be added to the soil in natural grassland compared to 2–5 Mg ha^-1^ through the leaf drop in trees each year [20].

Currently, SQIs are mostly based on so-called sum or black-box parameters and generally include microbial indicators such as microbial biomass, activity and biodiversity [13, 21, 22]. Recently, an alternative has been proposed, based on the use of specific ratios that report on function such as the quotients of microbial respiration-C-to-microbial biomass-C (*q*CO_2_) and the microbial biomass-C-to-Soil organic matter-C ratio (*q*Mic) [22, 23]. These indicators avoid the problems of comparing trends in soils with different organic matter or microbial biomass content and appears to provide a more sensitive indicator of soil changes than either activity or population measurements alone [23, 24]. In this study, we included these in the total data set (TDS) of 20 physico-chemical and biological soil properties. We used multivariate statistical methods to identify SQIs and explore the relationships between the SQIs and the soil type/vegetation cover. The selection of the 20 parameters for use in this study was based on the frequency of use of these parameters in literature (i.e. how often the parameters appear in scientific papers) and their availability. Using factor analysis the 20 variables were reduced to 6 uncorrelated factors (linear functions) also called soil quality factors or SQIs. The specific objectives of the study were to: (1) identify appropriate soil SQIs in soils of Great Britain, (2) determine whether soil types (conventional soil classifications) or aggregate vegetation classes (AVCs) could predict SQI and (3) determine to what extent do soil types and/ AVCs may act as major regulators of SQI.

## Materials and methods

### Soil sampling and preparation

Soil samples were collected throughout the Great Britain (GB; -England, Scotland and wales) as part of the Centre for Ecology and Hydrology Countryside Survey (CS) 2007 [25] with sites representing the main types of landscape and soil groups. To encompass all the major soil and land use types, a total of 304 soil samples were collected throughout the GB, based on a stratified random sample of 1 km squares at grid points on a 15 km grid using the Institute of Terrestrial Ecology (ITE) Land Classification (S1 Table/descriptions) as the basis of the stratification [26]. S1 Fig shows the general location and distribution of samples across Great Britain. At each grid intersection, a 1 km^2^ sample area was selected. Within the 1 km^2^ sample area, 3 plots (5 × 5 m^2^) were randomly located and a single 15 cm long × 4 cm diameter soil sample was collected from each of the plots. Top soils were only selected for sampling to reflect standard practice in national monitoring schemes [27] such as Soil Survey England and Wales handbook [28], the National Soil Monitoring Network [29] and the UK Soil Monitoring Network [30].

The soil leachate was collected according to Emmett et al. [31]. The soil leachate replicate cores were first wetted to field capacity with artificial rainfall (125 μM NaCl, 15.7 μM CaCl_2_, 1.3 μM CaSO_4_, 15.3 μM MgSO_4_, 12.3 μM H_2_SO_4_) in the dark at 10°C until the soils were fully wetted. The cores were then sprayed with artificial rainfall until a further 150 ml of leachate had been collected resulting in a solution with a pH of approximately 4.6. After washing out the cores, a small amount of suction was applied to drain larger pores. Cores were then incubated under anaerobic conditions for 4 weeks, at 10 ^0^C, approximately UK mean summer soil temperature Cores were then extracted with 1molar KCl, and ammonium and nitrate concentrations were determined as a measurement of mineralisable N using a TOC-VCSH/CSN analyser (Shimadzu Corp., Kyoto, Japan) as describe below.

Across all land uses and aggregate vegetation class (AVC) categories, the dominant soil types (% of total) were: brown soils (32%), groundwater gleys (13%), surface water gleys (19%), lithomorphic soils (8%), peats (15%), pelosols (2%) and podzolic soils (11%) (S2 Table). See S2 Table for their equivalents in the WRB classification. All the sites were characterised by a temperate climate with a North-South mean annual temperature range of 7.5 to 10.6°C and East-West mean annual rainfall range of 650 to 1700 mm.

### Aggregate vegetation classes

The vegetation data from the plots were analysed using the classification by Aggregate Vegetation Classes (AVCs). The AVCs were the vegetation types produced from a quantitative hierarchical classification of the different species found in sample plots. The eight AVCs used for assessing vegetation condition are listed in Table 1. Across all the soils sampled, the AVCs represented (% of the total): 18% crop and weeds, 17% fertile grasslands, 22% heath and bogs, 20% infertile grasslands, 2% lowland woodland, 10% moorland grass mosaics, 4% tall grass and herbs and 7% upland woodland.

**Table 1:** Summary of the aggregate vegetation classes (AVCs) used for assessment of vegetation condition.

Aggregate vegetation class (AVC) +(abrev)	Description
1. Crops and weeds (Craw)	Weedy communities of cultivated and disturbed ground, including species-poor arable and horticultural crops.
2. Tall grass and herbs (Tgah)	Less intensively managed tall herbaceous vegetation typical of field edges, roadside verges, stream sides and hedge bottoms.
3. Fertile grassland (Frtg)	Agriculturally improved or semi improved grassland. Often intensively managed agricultural swards with moderate to high abundance of perennial rye grass.
4. Infertile grassland (Infg)	Less-productive, unimproved and often species rich grasslands in a wide range of wet to dry and acid to alkaline situations.
5. Lowland wooded (Lwlw)	Vegetation dominated by shrubs and trees in neutral or basic situations, generally in lowland Britain. Includes many hedgerows.
6. Upland wooded (Uplw)	Vegetation of broadleaved and conifer woodland often in more acidic situations, generally in upland Britain.
7. Moorland grass mosaics (Mrgm)	Extensive, often unenclosed and sheep grazed hill pastures throughout Britain.
8. Heath and bog (Htab)	Vegetation dominated by heathers. Includes drier heaths as well as bog. Mostly in the uplands.

The brackets indicate the abbreviation of the vegetation class (adapted from Smart et al., [32].

### Soil analysis

Soil pH was determined in soil-distilled water extracts (1:2.5 w/v soil to water soil ratio) using a glass electrode (Gelplas general purpose electrode, BDH) and HI-209 pH meter (Orion research, Boston, MA, USA). Soil moisture was determined by weight loss after oven drying at 105°C overnight (>16 h). Water content at field capacity was estimated by saturating the soil followed by measuring the water retained in the soil at -33 kPa. Bulk density was calculated (mass/volume) on the oven dry soil after removal of stones from the cores (>2 mm in diameter). Soil organic carbon (SOC) was determined from loss on ignition (LOI). LOI was measured on a 10 g sub sample of oven dry soil by combusting at 375°C for 16 h. SOC was calculated according to the method of Ball [33] as:

SOC=(0.458×gLOI)‐0.4 [Eq 1]

Phosphorus was determined by the Olsen P method according to Emmett et al. [30]. Total C and N were determined using UKAS accredited method SOP3102 on an Elementar Vario-EL elemental analyser (Elementaranalysensysteme GmbH, Hanau, Germany) according to Emmett et al. [22, 31].

Soil respiration (SR) was determined on a 15 cm long, 2.5 cm diameter soil cores with a 1250 cm^3^ head space. The soils were incubated at 10°C for 1 h (at which linearity was established). Subsequently, the head space gas was analysed for CO_2_ concentration using a Clarus 500 Gas Chromatograph (Perkin Elmer Corp., Beverley, MA). The CO_2_ flux was established by comparing the CO_2_ concentration before and after incubation. Soil microbial biomass C and N were estimated on moist soil samples (10 g) using the modified chloroform-fumigation-extraction (CFE) method of Vance et al. [34]. For each soil 10g of the control and the fumigated samples were extracted with 1 M KCl. The TOC and TON in the 1 M KCl extracts was determined using a TOC-VCSH/CSN analyser (Shimadzu Corp., Kyoto, Japan). Extraction efficiency correction factors of 0.45 and 0.54 were used for microbial C and N, respectively [9, 35, 36]. Soil microbial biomass was therefore calculated according to the formula: C_mic_ = EC/*k*EC, where EC = (TOC in fumigated samples—TOC in control samples) and *k*EC = 0.45, and N_mic_ = EN/*k*EN, where EN = (total N in fumigated samples–total N in control samples) and *k*EN = 0.54. The microbial C:N ratios were subsequently calculated from these values.

The metabolic and microbial quotients were calculated indices. The metabolic quotient or coefficient was calculated as the ratio between the CO_2_-C from basal respiration and the microbial biomass-C (CO_2_-C_resp_-to-C_mic_), expressed as μg CO_2_-C mg^-1^ biomass-C h^-1^. It is also known as the specific respiration rate (*q*CO_2_) [37]. The microbial quotient was calculated as the ratio between the microbial biomass-C-to-total organic C (C_mic_-to-C_org_).

### Leachate analysis

Dissolved organic carbon (DOC) and dissolved organic nitrogen (DON) in leachate were measured using a TOC-VCSH/CSN analyser (Shimadzu Corp., Kyoto, Japan) and the DOC:TON ratio subsequently calculated. Nitrate and ammonium concentrations were measured with a Skalar SAN+^+^ segmented-flow autoanalyser (Skalar, Breda, Netherlands), based on the cadmium (Cd) reduction method [38, 39] and the modified Berthelot reaction [40] respectively. Electrical conductivity (EC) was measured with a standard platinum 1 cm electrode on a 4520-EC meter (Jenway Ltd, Dunmow, Essex, UK). pH was measured using a glass electrode (Gelplas general purpose electrode, BDH) on a HI-209 pH meter (Orion research, Boston, MA, USA). Total free amino acids were determined using the fluorometric OPAME procedure of Jones et al. [41] and a Cary Eclipse Fluorescence Spectrophotometer (Varian Inc., Australia) using a leucine standard. Humic substances were determined by measuring the absorbance of 350 μl of leachate at 254 and 400 nm (UV and visible range respectively) on a PowerWave XS scanning microplate spectrophotometer (BioTek^®^ Instruments, Winooski, VT, USA). The absorbance of deionised water was used as a control. A humification index (HIX) was calculated by dividing the absorbance at 254 nm by the absorbance at 400 nm [42, 43]. Soluble phenolic concentrations were assayed using a modification of the method of Box [44] and Ohno and First [45] using Na_2_CO_3_ (1.9 M) and the Folin-Ciocalteu reagent (Sigma-Aldrich, Poole, Dorset) [46]. The blue-coloured phenolics were measured at 750 nm using a PowerWave XS scanning microplate spectrophotometer (BioTek^®^ Instruments, Winooski, VT, USA).

### Statistical analyses

ANOVA, factor, discriminant and cluster analyses were all determined using SPSS version 15.0 (SPSS Inc., Chicago, IL) and GenStat version 8 (VSN International Ltd, Hemel Hempstead, UK). They were used to analyse the measured attributes to investigate the effect of soil types and AVCs on the SQIs identified. To identify significant differences between treatments, post hoc multiple comparison (pair-wise) tests were made using the Gabriel test where homogeneity of variance was assumed and Games-Howell procedure where unequal variance occurred. For the cluster analysis, the average linkage method and a squared Euclidean distance measure were used with a rescaled distance cluster combined measure on the similarity axis. The variables were standardized to minimize the effect of scale differences since the variables possessed different units.

## Results

### Biological, physical and chemical properties in the soils of Great Britain

**Fig 1:**
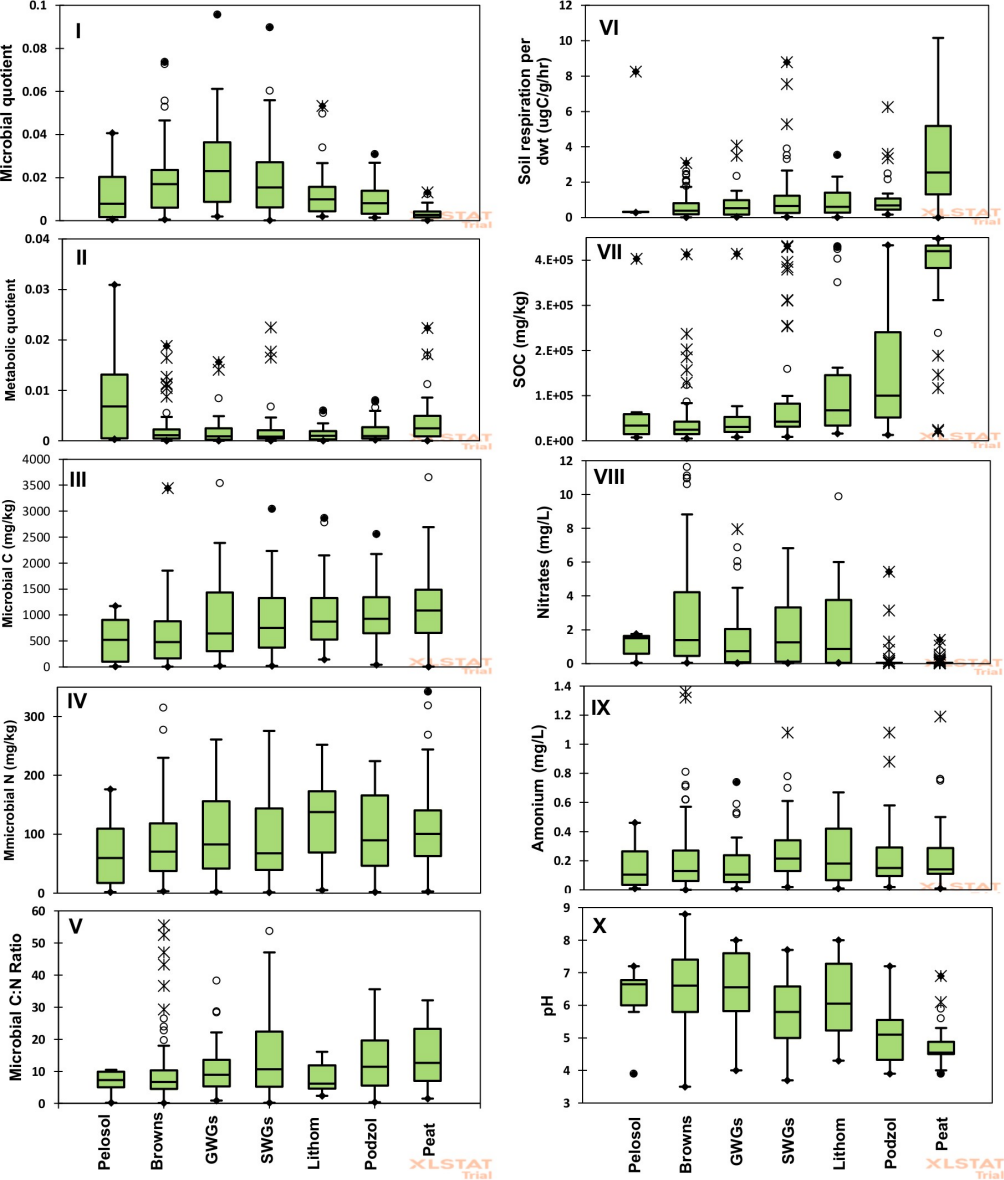
Box plots (panel I-X) showing the spread of each measured soil property for each of the major soil types from 304 individual soil samples. GWG and SWG represent groundwater and surface water gley soils respectively.

**Fig 2:**
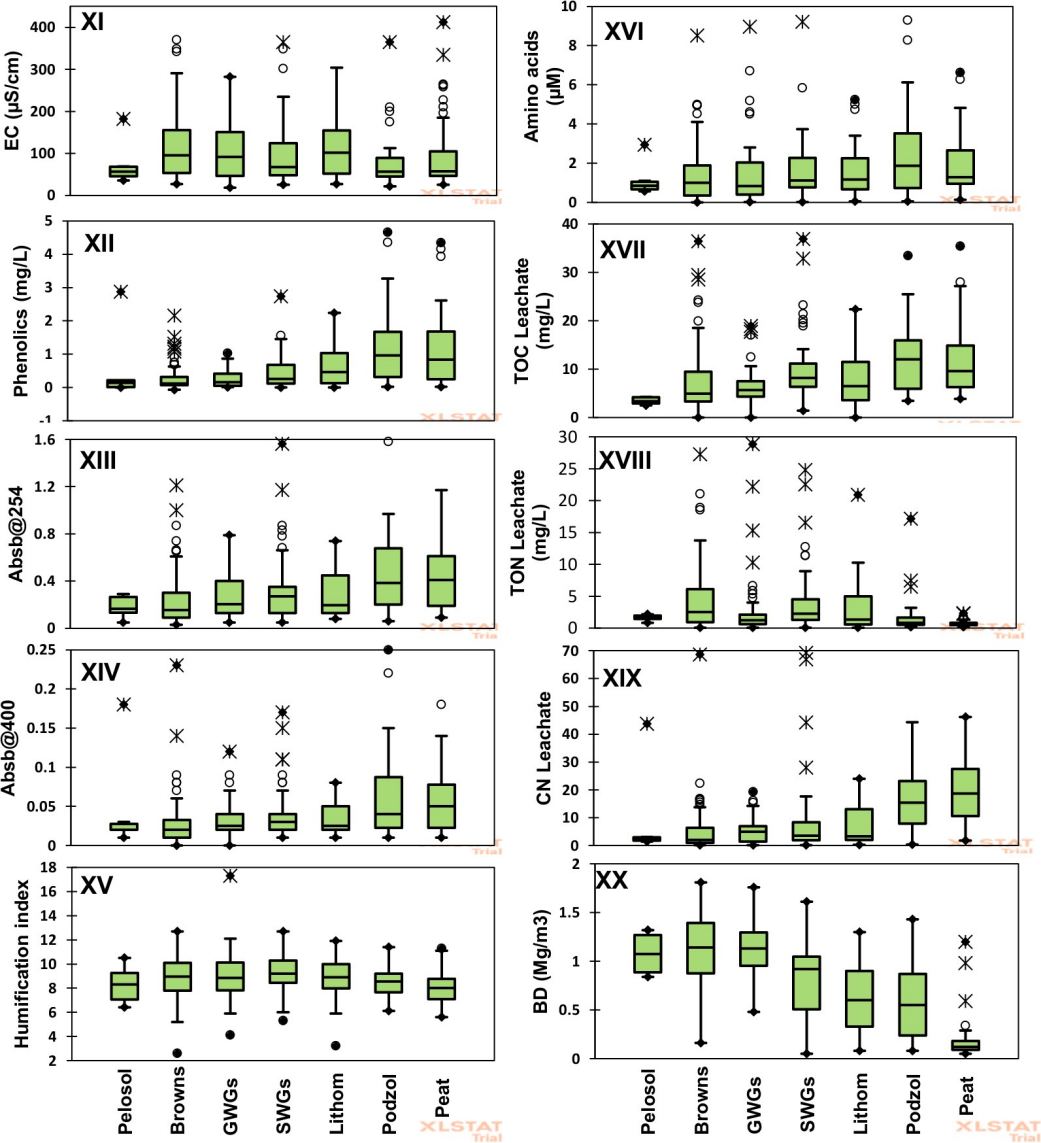
Box plots (panel XI-XX) showing the spread of each measured soil property for each of the major soil types from 304 individual soil samples.

The variability of individual soil quality indicators across the range of soil types is shown in Fig 1 (panel I-X) and Fig 2 (panel XI-XX). The box plots show the spread of each measured soil property for each soil type. From the box plots, most of the soil quality indicators did not show differentiations among the soil types save for the following: microbial quotient, SOC and soil respiration which separated the peats from the rest; pH and C:N leachate separated the peats and the podzols from the rest, while the bulk density grouped the soils in three groups of pelosols, the browns, ground-water gleys and the surface-water gleys (ave = 1.1Mg m^-3^) in one group; podzols and lithomorphics (ave = 0.5 Mg m^-3^) in the second group and peats (ave = 0.2 Mg m^-3^) in the third group. All other property values measured could not effectively differentiate the soil types.

### Relationships among soil properties

Correlation analysis of the twenty soil attributes representing soil biological, physical and chemical properties resulted in significant correlation (*P <* 0.05) in 112 of the 190 soil attribute pairs (Table 2). Of these, the highest significant (*P* < 0.01) positive correlations was between humic substances at 254 nm versus those at 400 nm (*r* = 0.97). Other highly significant (*P* < 0.01) positive correlations were between the absorbance at 254 nm or 400 nm versus DOC (*r* = 0.78 and *r* = 0.71 respectively); leachate DON versus NO_3_^-^ (*r* = 0.78), and bulk density versus pH (*r* = 0.70). Additional notable significant (*P* < 0.01) positive correlations (*r* > 0.50) were between: microbial-N versus microbial-C, SOC versus soil respiration, the leachate C:N ratio versus SOC, electrical conductivity versus both nitrate and DON, phenolics versus absorbance at 254 nm and DOC versus absorbance at 400 nm. The highest significant (*P* < 0.01) negative correlation was between bulk density versus SOC (*r* = - 0.83). Other notable significant (*P* < 0.01) negative correlations were between: bulk density versus either microbial biomass C (*r* = -0.42), soil respiration (*r* = -0.51) or the leachate C:N ratio (*r* = -0.47); SOC versus *q*Mic (*r* = -0.47) and pH versus either SOC (*r* = -0.66), UV-visible absorbance spectroscopy at 400 nm (*r* = -0.42), leachate DOC (*r* = -0.40) or leachate C:N ratio (*r* = -0.47).

**Table 2:** Correlations among physical, chemical and biological soil attributes.

**Variable**	**qMic**	**qCO2**	**Mic C**	**Mic N**	**Mic CN**	**SR**	**SOC**	**Nitrate**	**Ammonium-N**	**pH**
**qMic**	1									
**qCO2**	-0.07	1								
**Mic C**	0.23**	-0.07	1							
**Mic N**	0.17**	-0.05	0.63**	1						
**Mic CN**	0.18**	-0.02	0.03	-0.24**	1					
**SR**	-0.26**	-0.01	0.31**	0.09	-0.02	1				
**SOC**	-0.47**	-0.04	0.39**	0.09	0.04	0.61**	1			
**Nitrate-N**	0.21**	-0.01	-0.15**	-0.12*	0.20**	-0.22**	-0.33**	1		
**Ammonium-N**	-0.05	-0.04	0.08	0.06	-0.03	0.02	0.04	0.06	1	
**pH**	0.35**	0.08	-0.31**	0	-0.11*	-0.39**	-0.66**	0.25**	-0.18**	1
**Ec**	0.03	0.06	-0.09	0.01	0.17**	-0.08	-0.03	0.59**	0.03	0.12*
**Phenols**	-0.23**	-0.01	0.19**	0.08	-0.01	0.27**	0.39**	-0.19**	0.38**	-0.36**
**Absb @ 254**	-0.24**	-0.01	0.10*	-0.03	0.05	0.22**	0.34**	-0.19**	0.23**	-0.42**
**Absb @ 400**	-0.23**	-0.01	0.10*	-0.06	0.05	0.21**	0.35**	-0.19**	0.23**	-0.42**
**HIX**	0.06	0.02	0	0.11*	0.13**	-0.07	-0.14**	0.24**	0.01	0.10*
**amino acids**	-0.04	-0.03	0.09	-0.04	0.28**	0.03	0.11*	-0.02	0.48**	-0.15**
**DOC_L**	-020**	0.01	0.12*	-0.02	0.06	0.29**	0.35**	-0.18**	0.32**	-0.40**
**DON_L**	0.18**	0.02	-0.08	-0.05	0.24**	-0.14**	-0.21**	0.78**	0.11*	0.09
**CN_L**	-0.25 **	-0.04	0.16**	-0.02	0.03	0.33**	0.50**	-0.33**	-0.04	-0.47**
**BD**	0.46**	0.05	-0.42**	-0.22**	-0.07	-0.51**	-0.83**	0.35**	-0.14**	0.70**
**Variable**	**Ec**	**Phenols**	**Absb @ 254**	**Absb @ 400**	**HIX**	**amino acids**	**DOC_L**	**DON_L**	**CN_L**	**BD**
**Ec**	1									
**Phenols**	0.04	1								
**Absb @ 254**	-0.04	0.58**	1							
**Absb @ 400**	-0.08	0.60**	0.97**	1						
**HIX**	0.37**	-0.09	-0.04	-0.22**	1					
**amino acids**	-0.01	0.23**	0.09	0.09	0.11*	1				
**DOC_L**	0.01	0.56**	0.78**	0.71**	0.08	0.23**	1			
**DON_L**	0.66 **	-0.08	-0.13**	-0.14**	0.31 **	0.05	-0.05	1		
**CN_L**	-0.05	0.34**	0.38**	0.37**	-0.06	0.02	0.38**	-0.25 **	1	
**BD**	0.10*	-0.38**	-0.35 **	-0.33**	0.04	-0.17 **	-0.37**	0.21**	-0.4**	1

Note

*Correlation is significant at *P* < 0.05 level, and

** at the *P* < 0.01 level; *q*Mic, microbial quotient; *q*CO_2_, metabolic quotient; Mic C, microbial biomass C (mg C kg^-1^); Mic N, microbial biomass N (mg C kg^-1^); Mic C:N, microbial biomass C:N ratio; SR, soil respiration (mg C kg^-1^ h^-1^); SOC, soil organic carbon (mg C kg^-1^); NO_3_^-^, nitrate N (mg N l^-1^); NH_4_^+^, ammonium N (mg N l^-1^); EC, (μS cm^-1^); Phenols, Soluble phenolics (mg l^-1^); Abs @ 254 and 400, UV-visible absorbance spectroscopy of soil solution at 254 and 400 nm; HIX, humification index; Am acids, Free amino acids (μM); DOC/N -L, dissolved organic carbon/nitrogen in leachate (mg l^-1^); BD, bulk density.

### Extraction and identification of factors or SQIs

Due to differences in the units of individual variables, factor analysis (FA) was performed using a correlation matrix on the standardised values of the measured 20 attributes. The generalised least-squares method was used to extract factors because it is robust and requires no assumptions of sample coming from a multivariate normal distribution [47]. The first six factors with eigenvalues > 1 were retained for interpretation, whilst factors with eigenvalues < 1 explained less total variation than individual soil attributes [48]. The retained factors accounted for > 61% of the total variance in the measured attributes; see Table 3.

**Table 3:** Total variance (eigenvalue), proportion and cumulative variance (prop var and cum var) explained by factor analysis using correlation matrix (standardized data) on the measured attributes.

Factors	Initial eigenvalues			Extraction sums of squared loadings			Rotation sum of squared loadings		
	Total	Prop of Var (%)	Cum Var (%)	Total	Prop of Var (%)	Cum Var (%)	Total	Prop of Var (%)	Cum Var (%)
Factor 1	5.31	26.6	26.6	3.60	18.0	18.0	3.35	16.7	16.7
Factor 2	2.64	13.2	39.8	3.22	16.1	34.1	2.96	14.8	31.5
Factor 3	2.03	10.1	49.9	2.14	10.7	44.8	2.28	11.4	42.9
Factor 4	1.73	8.7	58.6	1.56	7.8	52.6	1.65	8.3	51.2
Factor 5	1.31	6.6	65.1	0.65	3.3	55.9	1.32	6.6	57.8
Factor 6	1.18	5.9	71.1	1.15	5.7	61.6	0.76	3.8	61.6

The retained factors were subjected to a varimax rotation. A varimax rotation redistributes the variance of significant factors and minimizes the number of variables that have high loadings on each factor, thereby simplifying the interpretation of the factors [47]. The relative importance of each soil attribute, in terms of its contribution to all of the factors, was judged by its communality value [49, 50] and is shown in Table 4. The six factors explained > 90% variance in UV-visible absorbance spectroscopy @ 254 and 400 (absb@254 and 400), microbial biomass carbon (Mic C), and soil organic carbon (SOC); > 80% in dissolved organic nitrogen in leachate (DON_L) and bulk density (BD); > 70% in microbial biomass nitrogen (Mic N), nitrate N, ammonium N, electrical conductivity (EC), and total organic carbon in leachate (TOC_L); > 60% in microbial quotient (*q*Mic), pH and humification index (HIX); > 50% microbial biomass C/N ratio (Mic CN), soil respiration (SR), and phenolics; and < 50% C/N ratio of the leachate (CN_L) and microbial metabolic quotient (*q*CO_2_) (Table 4). Attributes with the low communality estimates (e.g. *q*CO_2_ and leachate C:N) were the least important for interpreting factors. The magnitudes of the loadings were used as a criterion for interpreting the relationship between the soil attributes and the factors. Soil attributes were assigned to the factor for which the loadings were highest.

**Table 4:** Proportion of variance (loadings) using varimax rotation and communality estimates for soil attributes of the retained factors.

Variable	Factor 1	Factor 2	Factor 3	Factor 4	Factor 5	Factor 6	Communality extraction
*q*Mic	-0.54	-0.13	0.12	0.45	-0.01	-0.07	0.67
*q*CO_2_	0.05	-0.05	-0.05	-0.18	0.01	0.00	0.10
Microbial biomass C	0.29	0.03	-0.04	0.89	0.09	-0.03	0.90
Microbial biomass N	0.05	-0.04	-0.08	0.75	-0.02	0.14	0.73
Microbial biomass C:N	0.07	0.05	0.30	-0.03	0.11	0.01	0.51
Soil respiration	0.61	0.06	-0.07	0.09	0.03	-0.06	0.50
Soil organic C	0.92	0.16	-0.08	0.08	0.01	-0.06	0.91
Nitrate N	-0.27	-0.09	0.81	-0.04	0.01	0.04	0.77
Ammonium N	0.01	0.23	0.05	0.05	0.78	0.01	0.72
pH	-0.68	-0.28	0.02	-0.06	-0.18	0.05	0.68
Elec. conductivity	0.03	0.00	0.74	-0.03	-0.05	0.22	0.70
Soluble phenolics	0.29	0.52	-0.03	0.06	0.32	-0.10	0.55
Absorb @ 254 nm	0.17	0.98	-0.06	-0.01	0.04	0.03	1.00
Absorb @ 400 nm	0.17	0.96	-0.05	-0.02	0.04	-0.20	0.99
HIX	-0.06	-0.06	0.25	0.07	0.05	0.76	0.69
Amino acids	0.11	0.06	0.02	0.01	0.66	0.05	0.56
DOC (leachate)	0.24	0.71	-0.02	0.00	0.29	0.18	0.73
DON (leachate)	-0.12	-0.06	0.91	0.01	0.09	0.07	0.87
C:N (leachate)	0.47	0.26	-0.17	-0.02	-0.06	0.02	0.42
Bulk density	-0.86	-0.19	0.13	-0.18	-0.11	-0.05	0.87

The first factor explained 16.7% (see Table 3) of the total variance. It was named soil organic matter (SOM) because it had high positive loading for SOC (0.92), soil respiration (0.61) and leachate C:N ratio (0.47), a high negative loadings for bulk density (-0.86), pH (-0.68) and moderately on *q*Mic (-0.54). Grouping *q*Mic with the SOM factor rather than factor 4 was as a result of its stronger correlation with attributes constituting the SOM factor namely, soil respiration (*r* = -0.26), SOC (*r* = -0.47) and bulk density (*r* = 0.46) rather than with microbial biomass-C (*r* = 0.23) and microbial biomass-N (*r* = 0.17) of factor 4 (Table 3). The second factor explained 15% of the total variance with a high positive loading for soluble phenolics (0.52), leachate UV-visible absorbance spectroscopy at 254 nm (0.98), 400 nm (0.96) and leachate DOC (0.71) and consequently, was termed dissolved organic matter (DOM). The third factor explained 11% of the total variance with high positive loadings for nitrate N (0.81), leachate DON (0.91) and electrical conductivity (0.74) and was therefore termed soluble nitrogen factor. The fourth factor explained 8% of the total variance and had positive loadings for microbial biomass-C (0.89), microbial biomass-N (0.75) and a moderately loading for *q*Mic (0.45), and was termed microbial biomass. The fifth factor explained 6.6% of the total variance and had positive loading for ammonium-N (0.78) and amino acids (0.66) and was termed reduced N. The sixth factor explained only 4% of the total variance and had a high positive loading for HIX (0.76) and was termed DOM humification (DOMH).

### Effect of soil types on soil attribute means and factor scores

One way ANOVA revealed that most of the soil attributes and factors (indicators) scores varied significantly with soil types (Table 5). However, pairwise comparison showed that the effect of soil types on most attributes was very small. In most cases, only the peat soils were clearly significantly (*P <* 0.01) different from all the other soil types. Only SOM and microbial biomass factors (Factors 1 and 4 respectively) varied significantly (*P <* 0.05) with soil type. SOM factor mean scores were negative for brown, GWG, SWG and pelosol soils and positive for lithomorphic, peat and podzolic soils. Peats had the highest score and were significantly different from all other soil types on the SOM factor. Furthermore, peat soils had the highest SOC content to which the analysis also confirmed. The mean scores for SOM factor did not vary significantly (*P* > 0.05) among browns, GWGs and pelosols soils. Similarly, the lithomorphic, podzolic and SWG soils could not be differentiated by the SOM. The microbial biomass factor varied significantly (*P <* 0.05) between browns versus GWGs and lithomorphics only. Mean scores for DOM, soluble N, reduced N and DOM humification did not vary significantly (*P* > 0.05) among all soil types.

**Table 5:** Soil attribute means and factor scores in the different soil types.

Soil attributes	Soil types							SEM	ANOVA
	Brown	Groundwater gley	Lithomorphic	Peat	Pelosols	Podzolic	Surface water gley		
**Microbial quotient (ratio)**	0.018^a^	0.026^a^	0.014^ac^	0.003^b^	0.014^abc^	0.010^c^	0.018^a^	0.003	<0.01
***q*CO_2_** (ratio)	0.073	0.002	0.001	0.011	0.01	0.002	0.003	0.012	NS
**Microbial biomass-C (g kg^-1^)**	0.59^a^	1.00^ab^	1.03^ab^	1.37^b^	0.54^a^	1.02^ab^	0.89^ab^	0.13	<0.01
**Microbial biomass-N (g kg^-1^)**	0.085^a^	0.119^ab^	0.148^b^	0.113^ab^	0.071^ab^	0.111^ab^	0.099^ab^	0.016	0.03
**Microbial biomass C:N**	12.4	19.6	18.9	19.7	36.3	29.9	33.2	12	NS
**Soil respiration (mg kg^-1^ h^-1^)**	0.63^a^	1.10^a^	0.93^a^	3.35^b^	1.63^ab^	1.58^ab^	1.18^a^	0.45	<0.01
**Soil organic C (g kg^-1^)**	42^a^	45^a^	132^b^	377^c^	92^ab^	151^b^	98^b^	23	<0.01
**Nitrate (mg N l^-1^)**	3.00^a^	2.04^ac^	2.32^ac^	0.13^b^	1.13^c^	0.37^bc^	3.08^a^	0.39	<0.01
**Ammonium (mg N l^-1^)**	0.25	0.18	0.3	0.27	0.17	0.31	0.3	0.05	NS
**pH**	6.55^a^	6.56^a^	6.24^ac^	4.71^b^	6.18^ac^	5.08^b^	5.73^c^	0.2	<0.01
**Elect. conductivity (μS cm^-1^)**	129	107	124	99	74	81	116	16	NS
**Soluble phenols (mg l^-1^)**	0.33^ac^	0.26^a^	0.68^bc^	1.10^b^	0.56^abc^	1.20^b^	0.46^c^	0.16	<0.01
**Absorbance @ 254 nm**	0.25^a^	0.28^a^	0.29^ab^	0.47^b^	0.45^ab^	0.48^b^	0.32^ab^	0.48	<0.01
**Absorbance @ 400 nm**	0.028^a^	0.033^a^	0.032^ab^	0.061^b^	0.047^ab^	0.061^b^	0.036^ab^	0.009	<0.01
**Humification index (HIX)**	9.0^ab^	9.0^ab^	8.7^ab^	8.2^a^	8.3^ab^	8.6^ab^	9.3^b^	0.3	0.03
**Amino acids (μM)**	1.52	1.83	1.67	1.95	1.15	3.1	2.08	0.4	NS
**Leachate DOC (mg l^-1^)**	7.5^a^	6.9^a^	8.2^ab^	12.0^b^	12.8^ab^	12.3^b^	9.8^ab^	2.2	<0.01
**Leachate DON (mg l^-1^)**	5.82^a^	3.47^ac^	3.16^ac^	0.78^b^	1.62^c^	1.81^bc^	6.69^a^	0.8	0.01
**Leachate C:N**	4.6^a^	5.5^a^	7.2^a^	19.0^b^	9.1^ab^	17.5^b^	9.7^a^	2.4	<0.01
**Bulk density (g cm^-1^)**	1.10^a^	1.11^a^	0.63^b^	0.19^c^	1.08^a^	0.58^b^	0.81^b^	0.06	<0.01
**Factors**	**Factor scores**								
**Factor 1** (SOM)	-0.52^a^	-0.63^a^	0.15^b^	1.58^c^	-0.59^a^	0.2^b^	-0.07^b^	0.12	0
**Factor 2** (DOM)	-0.17	-0.05	-0.13	0.22	-0.46	0.44	0	0.15	NS
**Factor 3** (Soluble-N)	0.09	-0.1	-0.06	-0.13	-0.4	-0.28	0.23	0.11	NS
**Factor 4** (Microbial biomass)	-0.24^a^	0.36^b^	0.30^b^	0.03^ab^	-0.21^ab^	0.01^ab^	0.02^ab^	0.19	0.04
**Factor 5** (Reduced-N)	-0.05	-0.22	-0.03	-0.11	-0.2	0.36	0.14	0.17	NS
**Factor 6** (DOMH)	0.06	-0.1	0.17	-0.3	-0.29	-0.2	0.23	0.18	NS

Superscripts letters indicate significant between soil groups at P<0.05 level.

### Prediction of soil quality indicators across soil types

Discriminant analysis of the six statistical factors in relation to soil types, indicated that the SOM was the most powerful in discriminating among the seven soil type groups based on the magnitude of their discriminant coefficients (Eq 2). The first canonical discriminant function explained 90% of the total variance based on Wilks’s Lambda, (*P <* 0.001) and therefore was the most important canonical discriminant function for discriminating soil types using the soil quality factors identified. Although the second canonical discriminant function was also significant (*P* = 0.03), it accounted for only 4% of the total variance and was therefore not used.


$$Y_{1}=1.43\;(SOM)+0.29\;(DOM)+0.08\;(microbial\;biomass)+0.03\;(reduced\;N)–0.14\;(soluble\;N)–0.22\;(DOMH)$$ [Eq 2]

Therefore the group differences across soil types shown by ANOVA can be explained in terms of SOM, judging from the discriminant coefficient which was five-fold larger than the coefficient for the DOM factor and several fold greater than the rest of the factors. Discriminant analysis of the measured attributes constituting to SOM i.e. microbial quotient (*q*Mic), soil respiration (SR), soil organic C (SOC), pH and bulk density (BD) indicated that *q*Mic was the most powerful attribute discriminating the soil types followed by BD (Eq 3).


$$Y_{2}=8.75\times 10^{‐6}\;(SOC)–1.99\;(qMic)–0.50\;(BD)–0.04\;(pH)–0.05\;(SR)$$ [Eq 3]

The discriminant coefficient for *q*Mic was four-fold larger than the coefficient for bulk density and more than 40-fold for the rest. The *q*Mic was significantly correlated with bulk density (0.46) at *P<0*.*01*, SOC (-0.47) at *P<0*.*01*, pH (0.35) at *P<0*.*01* and soil respiration (-0.26) at *P<0*.*01* while BD was significantly correlated with SOC (-0.83) at *P<0*.*01*, pH (0.70) at *P<0*.*01* and soil respiration (-0.53) at *P<0*.*01* meaning that *q*Mic and bulk density, though correlated, were the most important and dominant attributes for assessing soil quality across soil types. The mean comparisons using the Games-Howell approach indicated that the BD had similar discriminating power as the SOM factor among the soil types. *q*Mic mean values varied significantly with soil types separating peat < podzols < browns, GWGs and SWGs soils in increasing order (Table 5).

### Effect of aggregate vegetation class on factor scores and selected soil attributes

Aggregate vegetation class (AVC) showed more effects on factor scores than the soil types. The significant effects were observed in SOM, DOM, microbial biomass and DOMH. The soluble N and reduced N factors showed no significant variation among the AVCs (Table 6). The SOM factor had the highest mean factor scores (*P <* 0.01) in heath and bog. Mean scores between moorland grass mosaics and upland woodland were not significantly (*P* > 0.05) different; neither was it different (*P >* 0.05) among fertile grasslands, infertile grassland, lowland woodland and tall grass mosaic. The mean scores were lowest in crop and weeds and were significantly different (*P <* 0.01) from all other AVCs except in tall grass and herbs.

**Table 6:** Effect of Aggregate Vegetation Class (AVC) on factor scores and soil attribute means.

	Average vegetation class mean factor scores									
Factors	Crops & weeds	Fertile grasslands	Heath & bog	Infertile grassland	Lowland woodland	Moorland grass mosaics	Tall grass & herbs	Upland woodland	SEM	ANOVA
**Factor 1** (SOM)	-0.80^a^	-0.54 ^b^	1.43 ^c^	-0.50 ^b^	-0.40 ^b^	0.62 ^d^	-0.64 ^ab^	0.20 ^bd^	0.1	<0.01
**Factor 2** (DOM)	-0.40 ^a^	-0.11 ^ab^	0.30 ^b^	0.02 ^b^	0.41 ^ab^	-0.11 ^ab^	-0.06 ^ab^	0.51 ^ab^	0.19	<0.01
**Factor 3** (Soluble-N)	0.34	0.07	-0.09	-0.03	0.05	-0.34	0.12	-0.28	0.14	NS
**Factor 4** (Mic. biomass**)**	-0.49 ^a^	0.16 ^b^	0.07 ^b^	0.27 ^b^	-0.19 ^ab^	0.28 ^b^	-0.61 ^a^	-0.21 ^ab^	0.16	<0.01
**Factor 5** (Reduced-N)	-0.39	0.09	0.13	0.02	-0.12	0.22	-0.2	0.18	0.14	NS
**Factor 6** (DOMH)	-0.29 ^a^	0.04 ^ab^	-0.35 ^ab^	0.13 ^b^	1.15 ^c^	0.18 ^b^	0.38 ^bc^	0.63 ^bc^	0.17	<0.01
**Soil attributes**	**Soil attribute mean values**									
**SR (mg kg^-1^ h^-1^)**	0.29^a^	1.00 ^b^	3.22 ^c^	0.77 ^b^	0.67 ^ab^	1.44 ^b^	0.43 ^ab^	1.41 ^b^	0.23	<0.01
**SOC (g kg^-1^)**	16.7 ^a^	43.6 ^b^	350.2 ^c^	43.8 ^b^	46.4 ^b^	185.6 ^c^	25.0 ^ab^	119.8 ^c^	11.2	<0.01
**pH**	7.3 ^a^	6.4 ^b^	4.6 ^c^	6.3 ^b^	6.2 ^abd^	5.2 ^d^	6.6 ^ab^	4.7 ^dc^	0.2	<0.01
**BD (Mg m^-1^)**	1.37 ^a^	1.06 ^b^	0.21 ^c^	0.95 ^b^	0.89 ^b^	0.41 ^d^	1.22 ^ab^	0.48 ^d^	0.05	<0.01
***q*Mic (ratio)**	0.021^a^	0.023 ^a^	0.005 ^b^	0.021 ^a^	0.015 ^ab^	0.009 ^b^	0.015 ^ab^	0.010 ^ab^	0.003	<0.01

Superscripts letters indicate significant between soil groups at P<0.05 level.

Means scores for DOM factor varied significantly (*P <* 0.01) between crop and weeds verses herb and bog, and infertile grasslands; all other pairs did not vary significantly. For microbial biomass factor, crop and weeds and tall grass and herbs varied significantly (*P <* 0.01) against the fertile grassland, infertile grasslands, heath and bog, and moorland grass mosaics, while all other pairs were not significantly different (*P* > 0.05). The DOM humification factor showed that the mean scores varied significantly (*P <* 0.01) among crop and weeds versus infertile grassland and moorland grass mosaics versus lowland woodland only.

### Prediction of soil quality indicators across Aggregate Vegetation Classes (AVC)

The first canonical discriminant function of the discriminant analysis of the six factors across the AVCs explained 94% of the total variance (Wilks’s Lambda, *P <* 0.01) whose coefficients were used in the equation below:

$$Y_{3}=2.12\;(SOM)+0.49\;(DOM)+0.30\;(microbial\;biomass\;C)+0.36\;(reduced\;N)‐0.35\;(soluble\;N)‐0.20\;(DOMH)$$ [Eq 4]

From the discriminant coefficients in Eq 4 [Eq 4], SOM factor was the most powerful discriminating among the eight different AVCs. The SOM factor was more than four-fold larger than the coefficients of all others soil quality factors under consideration.

The discriminant analysis of the measured attributes constituting the SOM factor showed that BD and *q*Mic were the most powerful discriminating soil attributes among the seven AVCs [Eq 5].


$$Y_{4}=3.27\;(BD)+0.70\;(pH)+0.08\;(SR)–2.45\;(qMic)–2.75\times 10^{‐6}\;(SOC)$$ [Eq 5]

Bulk density possessed similar discriminating power as the SOM factor among the AVCs. Bulk density values were significantly different (*P <* 0.01) among AVCs with the lowest mean values in heath and bog (0.21 Mg m^-3^) < moorland grass mosaic (0.41 Mg m^-3^) and upland wooded (0.48 Mg m^-3^) < lowland wooded (0.89 Mg m^-3^), < infertile grass (0.95 Mg m^-3^), < fertile grass (1.06 Mg m^-3^), < tall grass and herbs (1.21 Mg m^-3^) and crop and weeds (1.37 Mg m^-3^; Table 6).

### Main and interactions effect of soil types and AVCs

The results of the two-way ANOVA on the first canonical discriminate function on all 20 variables showed significant (*P <* 0.01) main and interaction effects. The main effect of soil types and the effect of soil types * AVCs interaction on the attribute’s scores was very small (Partial Eta Square = 0.09 and 0.16 respectively), while the main effect of the AVCs was large (Partial Eta Square = 0.42; Table 7).

**Table 7:** Tests of between-subjects effects.

Source	Type IV sum of squares	Df	Mean Square	F	Sig.	Partial eta squared
Corrected model	553.14	38	14.56	36.36	0.001	0.844
Intercept	3.42	1	3.416	8.532	0.004	0.032
Soil Type * AVC_Desc	**18.98**	**25**	**0.759**	**1.896**	**0.008**	**0.157**
**Soil Type**	**10.36**	**6**	**1.726**	**4.311**	**0.001**	**0.092**
**AVC_Desc**	**73.97**	**7**	**10.57**	**26.39**	**0.001**	**0.420**
Error	102.09	255	0.400			
Total	655.24	294				
Corrected Total	655.24	293				

Notes: Dependent variable: 1st Canonical Discriminant function scores of all the soil attributes measured. AVC_desc means AVC description; Soil Type*AVC_Desc means the interaction between the soil type and AVC effects.

The cross tabulation of AVCs versus soil types (S3 Table), showed that 27 out of 56 combinations or cells, the soil types were sampled less than the calculated expected counts in the AVCs. In 16 combinations, the soil types were not at all represented in the AVCs. The most affected were the lowland woodland and tall grass and herbs where, only brown and SWGs were samples in the former and only browns, GWG and SWGs in the latter.

## Discussion

### Identification of SQI

A set of 20 correlated soil attributes were grouped into six factors called soil quality factors, using factor analysis. The factors identified contribute to one or more key soil functions and could be considered soil quality indicators [48, 51]. The SQI identified were SOM, DOM, soluble N, reduced N, microbial biomass, and DOMH with their associated attributes (namely SOC, *q*Mic, BD, pH, SR) for each category of groups (Soil types/AVC) (see Eqs 2–5). The discriminant analysis of these provides information on how sites differ with each other (if sites are similar in nature or not) and which SQIs and attributes contribute to site classification/discrimination [52]. These identified SQI are related to either the quantity of organic matter or its decomposition state and processes. This is because SOM is known to play vital roles in the maintenance and improvement of many soil properties and processes [53] and exerts a strong effect on soil function [54].

### Effect of soil types and AVCs on the factors or soil quality indicators

As we know, soil quality is a complex concept because it is determined by multiple parameters [19]. The soil quality factors are not measured directly [48, 55], therefore, the effect of soil types and the AVCs on these factors were inferred by analysing soil attributes that comprised them. Not all the soil quality factors varied significantly with soil types or with AVCs. Only SOM and microbial biomass factors varied significantly (*P <* 0.001) by soil types. SOM was able to discriminate the highest number of soil groups, separating the peats (1) with the highest scores, from lithomorphics, podzols, and SWGs (2) with intermediate scores, and from browns, GWGs and pelosols (3) with the lowest scores (Table 5), thus rendering three distinct soil type groupings. The microbial biomass factor had a minor effect, discriminating the browns from GWGs and lithomorphics only. The soil attributes constituting to these soil quality factors (SR, SOC, pH, BD, *q*Mic, microbial biomass C and N) showed significant (*P* < 0.01) variations discriminating at most three groups of soil types. In all the attributes considered, browns, GWGs and pelosols were grouped together. SOM factor, SOC and bulk density attributes separated the peats as a unique soil group from all other soil types, which is not entirely a surprising result, since the peats are highly organic in nature with low BD as opposed to mineral soils with low OM content and higher bulk densities. The most important soil quality indicator associated with specific soil types or groups was the SOM factor with *q*Mic > bulk density as the most important attributes.

Similarly, the most important Factors (SQIs) differentiating the AVCs across Great Britain was the SOM factor with bulk density > *q*Mic attributes being the most important attributes. Four distinct AVC groups were separated based on SOM factor and BD attribute. Heath and bog was exclusively separated as one group (1). Other groups were: (2) Crop and weeds with tall grass and herb; (3) Fertile grassland, infertile grassland, lowland woodland, tall grass and herbs, and upland woodland; (4) Moorland grassland mosaic with upland woodland. The upland woodland and tall grass and herbs were intermediate habitats classifying in more than one of these groups. The rest of the factors and attributes discriminated three or less groups. The soil attributes were generally better in discriminating the AVCs than the factors (SQIs) (Table 6)

Since *q*Mic and bulk density were moderately correlated (*r =* 0.46**), they may be redundant as indicators to be used together. If only one attribute were to be used to monitor soil quality in soil types and AVCs, *q*Mic and BD respectively seems to offers the greatest potential judging from their high weights on the respective prediction models. However the *q*Mic may be a ‘MUST be included’ soil attribute in the minimum data set, due to its important role in several soil functions, being a fraction of SOC. SOC influences a wide range of soil functions including bulk density, infiltration, pesticide buffering, aeration, aggregate formation, pH, buffer capacity, cation-exchange properties, mineralization, and the activity of soil organisms [51, 56, 57]. However, since the measurement of bulk density is reasonably easy to obtain, it is therefore reasonable to consider it together with SOC, microbial and biomass C as minimum data set for assessing soil quality across average vegetation classes in the study area.

Pedogenesis has taken place over thousands of years in the UK. During this period there has been a range of climate change related vegetation colonization phases starting from tundra heath and cycling through a range of forest types [58]. During this period parent material/topography, climate and vegetation would have been stable for long periods of time leading to the differentiation of soils. This was followed by progressive forest clearance which started approximately 1000–3000 years ago with vegetation cover becoming more grassland and heathland dominated. The last 200 years, however, has seen intense management of these soils with the addition of fertilisers, lime and organic wastes combined with mechanical mixing of the soils which has reversed centuries of acidification and soil horizon development. This homogenisation of the soil has led to shifts between soil types even over short timescales (e.g. humic-podzolic to brown soils on improved upland grasslands) and the loss of peat soils in intensive agricultural areas (e.g. East Anglia; [59]. One key question is therefore whether it is historical soil type or current vegetation that is more important in driving soil processes in the short term (e.g. over a 10–25 year timescale)? Here we found that more soil quality factors showed an AVC effect rather than a soil type effect. All soil quality factors varied significantly (*P* < 0.01) by AVCs except soluble N and reduced N factors though none discriminated more than four groups. Similarly, Peng et al. [19] and Guan and Fan [60] found strong evidence that different vegetation restoration types accounts for significant variations in the soil quality in different areas. It is possible that some of the soil quality factors that were insensitive to vegetation may represent inherent soil qualities that are controlled by other key factors of soil formation (e.g. parent material/topography), while those which significantly varied by AVCs may represent dynamic soil qualities, possessing great potential for assessing management practices on soil quality [4, 56, 61–63]. Most indicators available in literature have not been validated nor their sensitivity tested in a wide range of situations [64]. Some of the attributes measured and the soil quality indicators identified in this work are not usually used in the monitoring of soil quality, but are candidates for potential alternatives [22].

### Prediction of SQI by soil type or AVC

**Fig 3:**
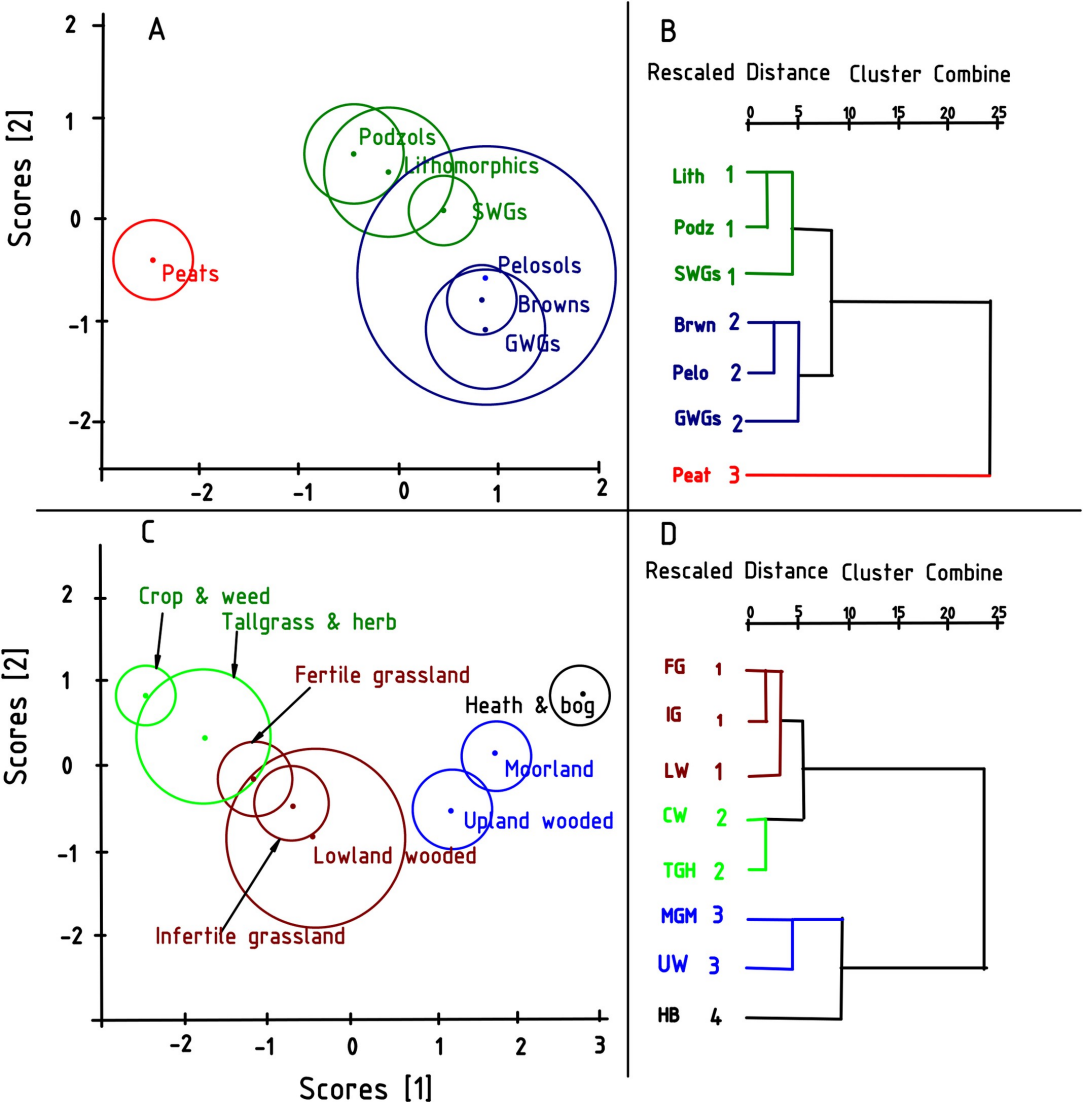
Discrimination plots showing 95% confidence circles around the means for soil types (Panel A) and AVCs (Panel C). Panels B and D are the respective cluster analyses dendrograms using a complete linkage method.

The question is: Can a combination of variables be used to predict group membership in soil type or AVC? The results from discriminant analysis was used to seek out a linear combination of SQI for each treatment group (soil type/AVC) that maximizes the difference between treatment groups for proper classification [52]. The analysis provides a classification function that determines to which groups an individual belongs. Eqs 2–5 shows the SQI and attributes that maximises the difference between groups in both the soil types and AVCs. The SQI identified were SOM, DOM, soluble N, microbial biomass, reduced N and DOMH with their associated coefficients and attributes for each category groups. The clusters from this multivariate classification are “natural” groups, which uses the “minimum-variance” solution; where a population is partitioned into cluster subsets by minimizing the total within group variation while maximising between groups variance [65]. The groups/cluster formed from the multivariate analysis need to have no significant overall spread. Even though SQIs were changes from one soil group to another [66], most of our cluster modes defined by soil types were not always distinct. Most of them were separated from each other by significant “noise” data, making it impossible to resolve all the clusters. Thus, the definition of the reference values for each soil type or AVC was ambiguous, since most soils types or AVC groups could not be differentiated (Fig 3). Forming, describing and defining the groups could involve the use all measured attributes even though only a few could be differentiating [67]. Even when a few soil quality factors/indicators and attributes were used, most of the groups/clusters could still not be resolved. From the discriminant plots and the dendrograms in Fig 3 three groups can be defined in soil types and four groups in the AVC.

An attempt to define unique property ranges in soil type groups could be based on bulk density attribute for the first group, a combination of SR and SOC attributes for the second group, and a combination of *q*Mic, soil respiration, SOC, pH and bulk density attributes for the third group. Nguemezi et al. [66] in their study also identified SOC and pH among the most influential soil attributes that differentiated soil groups their study area in Cameroon. The pelosols were the most dispersed and unreliable group in terms of attribute membership prediction, probably due to the fact that they were under sampled, considering that only six samples were included in the analysis. The classification of the AVCs using discriminate and cluster analyses on key attributes yielded four clusters. Defining differentiating criteria for these groups could involve the use of a combination of SR, SOC, and pH attributes to define property ranges for the first, second and fourth groups and bulk density attribute for the third group. Tall grass and herbs and lowland wooded were under sampled (with 11 and 6 samples respectively; S3 Table) which greatly compromised their predictive accuracy as can be observed from the large 95% confidence circles which overlapped with other AVC groups.

### To what extent do soil types and/ or AVC act as major regulators of SQI?

From the results, it’s clear that both soil types and AVCs regulate the physical, chemical and biological properties. The interaction of soil type × AVC (Table 7) reveal differences in their influence on the soil attributes. The ‘practical’ significance of each term from Partial Eta Square values indicates that AVCs (with a large Partial Eta Square = 0.42), were a better regulator of the SQIs than soil types (with a weak Partial Eta Square = 0.09). The effect size for the interaction was equally relatively weak (Partial Eta Square = 0.16). The conclusion of the significant (*P <* 0.01) interaction effect of soil type × AVC is that the soil type differences in the attributes partly depended on the AVCs where the soil was sampled. A multiple comparison of all soil type groups with AVC groups would be required to draw specific conclusions regarding the interaction effects, which is quite complex and is beyond the scope of this research. Suffice to say that there was a partial and varied soil type × AVC interaction across all levels. Correspondingly, studies by Setala et al. [68] and Xu et al. [69] reported similar interactions between soil types and vegetation types. They established that vegetation types, greatly modify the soil biogeochemistry of pH, %OM, %C, and %N and the C/N ratio, translating to significant variation in soil properties and processes among the vegetation types. In addition, Soil colour, pH, and electric conductivity (EC) are interlinked and controlled by the organic matter content of soil [70] and the quantity and quality of organic matter depends on the vegetation type. Therefore, soil organic matter is one of the most important determinants of soil quality and has a close association with soil productivity and fertility [71]. These interactions results are in accordance with Jenny’s [62] theory of soil formation which states that the biotic factor (of which vegetation plays a major role) is amongst others an important soil forming factor. However, the results from the cross tabulation indicated that not all soil types were well represented in the AVCs going by the calculated expected counts. In some cases, soil types were not at all represented (S3 Table). This problem can contribute to the complexity and diminished accuracy in the interpretation of the interaction effect observed above.

## Conclusions

The SQIs or attributes that contribute to the site discrimination in GB environment were identified as SOM, DOM, soluble N, microbial biomass, reduced N and DOMH with their associated attributes. The dominant SQIs from factor analysis or attributes varied by both soil type and AVC of which the SOM factor was the most discriminating factor. The *q*Mic and BD were the most discriminating measured attributes which produced three fairly homogenous groups for soil types and four groups for AVCs. However, it was impossible to define reference values in the SQIs or attributes for separate individual soil types or AVCs, as property ranges greatly overlapped due to large between group variability (probably due to integrating large spatial areas). This suggests that soil types or AVCs are poor predictors for SQIs across different regions of differing climatic conditions and edaphic factors making it impossible to select a universal optimum set of indicators that define them. Localised areas with similar climatic and topoedaphic factors may hold promise for the definition of SQI that may predict the soil types as some of the differences observed in soil types (with regard to soil attributes) were in part dependent on the AVCs differences. A few workers [2, 48, 50, 63, 64, 67] in different regions have attempted to define sets of SQI with reasonable success.

For further research, it might be worthwhile to make special consideration for the climatic, spatial and parent material variability in the sampling designs in addition to the inclusion of other promising soil attributes. The sampling design, should ensure equal and adequate representation of soil types in the aggregate vegetation classes in order to accurately capture the interaction effect. Other key soil quality indicators, may include measures of key soil enzymes (e.g. cellulase, protease, phosphatase, sulfatase), their potential to release N_2_O and CH_4_.

## Supporting information

S1 Fig Map of the Great Britain, UK. Soil sampling locations used in the study. The total land area is 209,331 km^2^.
(TIF)

S1 Table Land class classification/descriptions. The land class classification with the corresponding land uses.
(DOCX)

S2 Table Shows conceptually comparable classification of the soils in the World Reference Base (WRB) classification. Number in brackets indicates the number of samples for that soil type.
(DOCX)

S3 Table The cross tabulation table of aggregate vegetation classes (AVCs) versus soil types. (DOCX)

S1 Dataset The minimum dataset for the study. (XLS)
