# Supplementary figures and images for: Identification and predictability of soil quality indicators from conventional soil and vegetation classifications
Source: PLoS One. 2021 Oct 22;16(10):e0248665. doi: 10.1371/journal.pone.0248665 (PMC8535190; doi:10.1371/journal.pone.0248665)

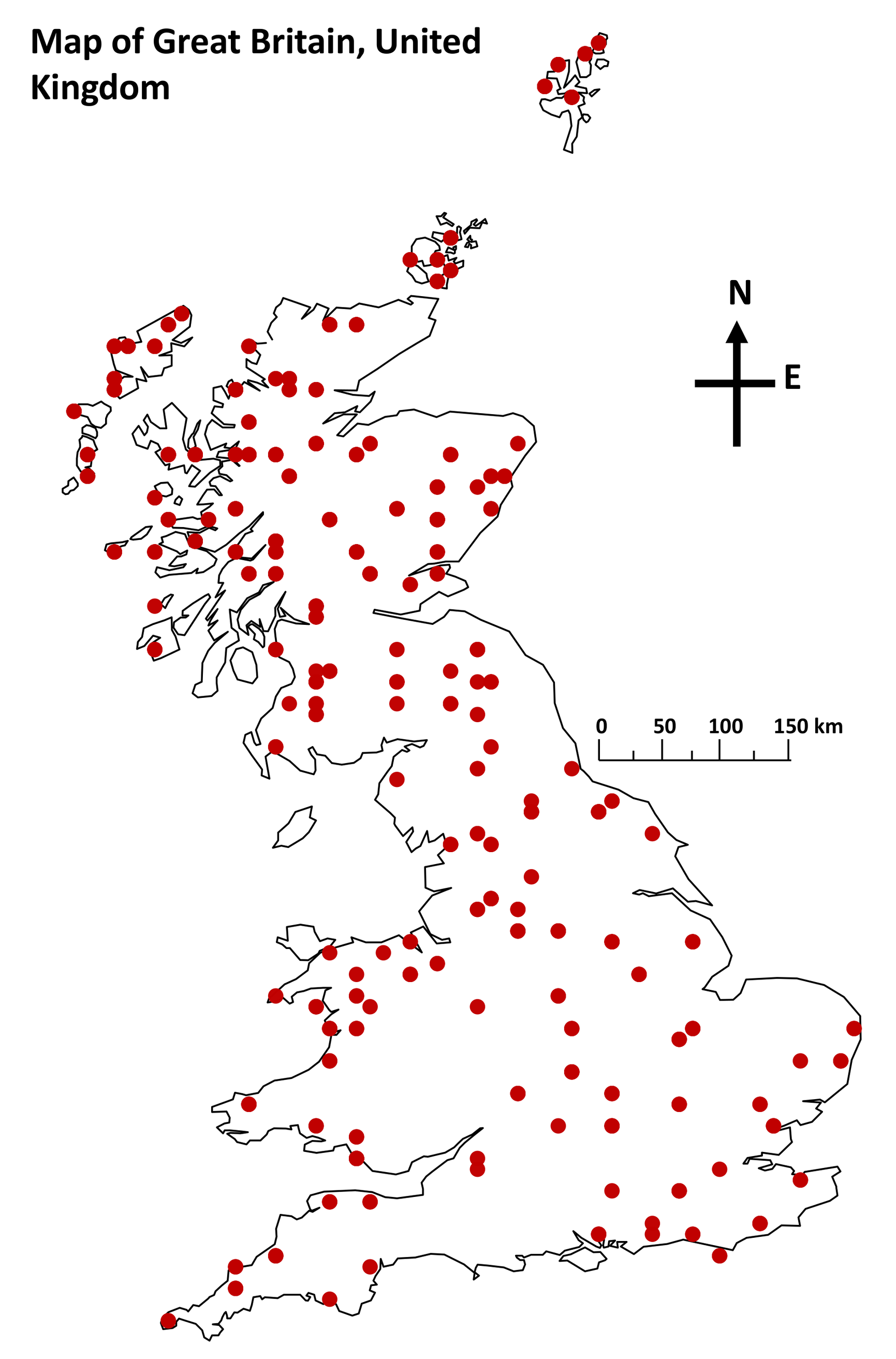

Supplement: S1 Fig — Soil sampling locations used in the study. The total land area is 209,331 km2. (TIF) [file pone.0248665.s001.tif]
