# Supplementary material for: Identification and predictability of soil quality indicators from conventional soil and vegetation classifications
Source: PLoS One. 2021 Oct 22;16(10):e0248665. doi: 10.1371/journal.pone.0248665 (PMC8535190; doi:10.1371/journal.pone.0248665)
Supplement: S1 Table — The land class classification with the corresponding land uses. (DOCX) [file pone.0248665.s002.docx]

**S1 Table/List: Institute of Terrestrial Ecology (ITE) Land Classification Descriptions**

**Descriptions of Land Classes with the corresponding land uses**

LAND CLASS: ONE

Geography: S. Wales, S.W. England, S. England.

Land form: Alluvial plains, low ridges, or plateaus with little surface drainage.

Topography: Gently rolling country or almost flat country mainly at medium to low altitude.

Landscape: Varied lowland landscapes with hedges, trees and farm buildings.

Land use: Cereals, good grasslands and limited native vegetation.

Soils: Mainly brown earths but also gleys.

Vegetation: Limited but grassland where present.

LAND CLASS: TWO

Geography: S. England, S.W. Midlands.

Land form: Downland summits and scarps, low ridges or occasionally alluvial plains.

Topography: Sweeping curves or smooth slopes with land at medium low or low altitudes.

Landscape: Mainly open or wooded downland with few hedges and scattered farmhouses,

Land use: Mainly good grassland but extensive cereals and built upland.

Soils: Brown earths or calcareous brown earths.

Vegetation: Rough grassland or bracken where present.

LAND CLASS: THREE

Geography: E. Anglia, S.E. England.

Land form: Alluvial plains or shallow river valleys with low broad ridges.

Topography: Flat or almost flat with virtually all land at low altitude.

Landscape: Prairie type lowlands with intensive agriculture and declining hedges,

Land use: Cereals, other crops and short term grassland.

Soils: Gleys, calcareous brown earths and brown earths.

Vegetation: Virtually absent.

LAND CLASS: FOUR

Geography: E. Anglia margins, S. England, S. Midlands.

Land form: Fenland or flood plains with intricate drainage patterns.

Topography: Flat or virtually flat, almost entirely at low altitude.

Landscape: Intensively farmed lowlands often under urban pressure.

Land use: Arable, with cereals and other crops, good grassland and urban.

Soils: Gleys with some calcareous brown earths.

Vegetation: Virtually absent.

LAND CLASS: FIVE

Geography: S. England, S.W. England, S.W. Midlands, S. Wales.

Land form: Variable from scarpland to downland and valley floors.

Topography: Uniform gentle slopes or smooth outlines mostly at low altitude.

Landscape: Varied lowlands with many natural features.

Land use: Mixed farmland although predominantly good grass; much urban,

Soils: Gleys and brown earths predominate.

Vegetation: Limited but varied where present from bracken to rushes.

LAND CLASS: SIX

Geography: S.W. England, S. Wales and S.W Midlands.

Land form: Dissected tablelands and plateaus with many small rivers.

Topography: Complex with many broad even slopes and the majority of land at medium/low altitude

Landscape: Intricate with small fields enclosed by hedges on banks with small woodlands.

Land use: Mainly good grassland but with some barley.

Soils: Brown earths and gleys predominate.

Vegetation: Limited to small areas.

LAND CLASS: SEVEN

Geography: S. England, S.W. England and Wales coasts.

Land form: Variable coastal morphology, mainly cliffs cut into tablelands.

Topography: Usually coastal cliffs, rarely estuarine, most land low altitude.

Landscape: Varied coasts backed by lowland farmland with farm houses.

Land use: Mainly pasture with some arable and good grass.

Soils: Brown earths but also other types.

Vegetation: Limited, but varied particularly moorland and grassland types.

LAND CLASS: EIGHT

Geography: E. Anglia, S. England, Wales, N.W. England coasts.

Land form: Marine alluvial plains bordering estuaries or rarely rocky coasts.

Topography: Mainly flat hut with some steeper coasts, most land low altitude.

Landscape: Usually flat coasts backed by good farmland effected by urban development.

Land use: Mainly pasture but some arable, extensive mudflats and urban development.

Soils: Gleys and brown earths.

Vegetation: Limited, but rough grassland where present.

LAND CLASS: NINE

Geography: N. Midlands, N.E. England, and S.E. Scotland.

Land form: Mainly valley floors and flood plains of large rivers together with bluffs.

Topography: Almost flat or gently rolling, most land medium/low altitude,

Landscape: Open lowland country often with declining hedges, intensive agriculture.

Land use: Mixture of good grass and arable with many urban areas.

Soils: Brown earths, gleyed brown earths and gleys.

Vegetation: Very limited, bracken or rough grassland where present.

LAND CLASS: TEN

Geography: N. Midlands, N.E. England, and S.E. Scotland.

Land form: Mainly valley floors or alluvial plains often with moderate scarps on margins.

Topography: Gentle slopes, often long with the majority of land medium/low but also low altitude.

Landscape: Well-farmed lowland country with many hedgerows and small woods.

Land use: Mainly arable but with good grassland and pasture also widespread.

Soils: Gleys with some brown earths.

Vegetation: Very restricted.

LAND CLASS: ELEVEN

Geography: E. and C. Midlands.

Land form: Alluvial plains or low broad ridges drained by small streams.

Topography: Very gradual slopes or flat with almost all land at low altitude.

Landscape: Open landscapes with large fields and declining hedgerows.

Land use: Arable predominates particularly wheat with good grassland and urban.

Soils: Gleys and brown earths.

Vegetation: Very restricted,

LAND CLASS: TWELVE

Geography: E. Midlands and Fens.

Land form: Mainly fens or flood plains and large rivers otherwise graded ridges.

Topography: Flat or almost flat entirely at low altitude.

Landscape: Prairie landscapes with derelict hedges and urban development.

Land use: Arable, mainly wheat with limited good grassland and urban.

Soils: Gleys and brown earths.

Vegetation: Virtually absent.

LAND CLASS: THIRTEEN

Geography: N. Wales, N.W. England, S.W. Scotland.

Land form: Heterogeneous, from low ridges in alluvial plains to scarps and river valleys.

Topography: Smooth slopes, rarely steeper almost entirely at low altitudes.

Landscape: Varied lowland landscapes with hedged small fields often affected by urban.

Land use: Usually mixtures of arable and good grassland but also variety of other uses.

Soils: Gleys and brown earths predominate but other types often present.

Vegetation: Bracken and rough grassland, but also some moorland.

LAND CLASS: FOURTEEN

Geography: N.W. and N.E. England, S.W Scotland.

Land form: Mainly marine or alluvial flood plains bordering estuaries, rarely rocky coasts.

Topography: Fiat or gently sloping with the majority of land at low altitude.

Landscape: Prairie landscapes with fences or neglected hedges much affected by urban development.

Land use: Mainly arable but also good grassland and much urban.

Soils: Gleys, gleyed brown earths and brown earths.

Vegetation: Very little present.

LAND CLASS: FIFTEEN

Geography: Wales, N. England.

Land form: Variable from dissected plateaus to valley floors bordered by escarpments,

Topography: Complex with shallow or occasionally steep slopes, flat land almost entirely medium/low altitude.

Landscape: Intricate lowland landscapes with many natural features.

Land use: Mainly pasture mixed with good land and arable.

Soils: Brown earths, gleys and some brown podzolics.

Vegetation: Restricted but mainly rough grassland and some bracken.

LAND CLASS: SIXTEEN

Geography: N. England, S.W, Scotland.

Land form: Flood plains or valley floors with escarpments or gently folded.

Topography: Mainly undulating land with some flat areas mainly at low altitudes.

Landscape: Varied lowland, well-farmed landscapes with many hedges.

Land use: Varied with mixtures of arable pasture and good grassland.

Soils: Brown earths and gleys.

Vegetation: Varied but with grassland types predominating and some moorland.

LAND CLASS: SEVENTEEN

Geography: S.W. England, Wales, N. England.

Land form: Plateaus or tablelands, with scarps often dissected by small rivers.

Topography: Some gentle slopes, but mainly quite steep hillsides at medium/high altitude.

Landscape: Open or enclosed marginal uplands with walls, fences and occasional farmhouses,

Land use: Mainly pastures with some good grassland.

Soils: Brown earths and brown podsolics but a range of other soils.

Vegetation: Mainly rough grassland types but also some moorland.

LAND CLASS: EIGHTEEN

Geography: Wales, N. England, W. Scotland.

Land form: Glaciated river valleys with steep scarps backing onto tablelands or distinct mountains.

Topography: Steep hillsides predominate with some more moderate slopes mainly at medium high altitudes.

Landscape: Mainly open, rugged uplands but with some areas transitional to enclosed land.

Land use: Predominantly rough grazing with some limited pasture land.

Soils: Brown podsolics, brown rankers, peats and other upland types.

Vegetation: Mainly moorland with extensive peatland and montane grassland,

LAND CLASS: NINETEEN

Geography: N. England, S. Scotland.

Landform: Broad ridges or flat topped or rounded summits with small rivers with flat floor.

Topography: Mainly moderately steep slopes but also some rather steep hillsides at medium high altitudes.

Landscape: A mixture of enclosed upland but also open mountains often afforested.

Land use: Mainly rough grazing or forest but some pasture.

Soils: Varied upland type but brown earths, podsols and peats the most abundant.

Vegetation: Mainly moorland but also mountain grass and peat types,

LAND CLASS: TWENTY

Geography: N. England, S. Scotland.

Land form: River valleys often with subsidiaries and scarps backing onto rounded hills.

Topography: Often complex including steep hillsides and more moderate gradients at medium/high altitudes.

Landscape: Mixtures of upland and marginal lowland with fences and walls.

Land use: Much pasture but some good grassland and occasional crops.

Soils: Gleys and brown earths with some other upland types.

Vegetation: Mainly rough grassland types but some peatland also.

LAND CLASS: TWENTY-ONE

Geography: C. and N. Scotland.

Land form: Peneplain surfaces with complex drainage or broad ridge with indistinct summits.

Topography: Predominantly quite steep hillsides but also some more moderate slopes

Landscape: Bleak upland landscapes, sometimes enclosed by walls or fences and afforested.

Land use: Open range grazing or forest.

Soils: Peats, peaty gleys or podsols.

Vegetation: Moorland or peatland types with some rough grassland.

LAND CLASS: TWENTY-TWO

Geography: N. England, S., C. and N. Scotland.

Land form: Dip slopes of plateaus or broad glacial valleys leading to rounded summits.

Topography: Slopes of variable gradient from steep to moderate and almost entirely at medium/ high altitudes.

Landscape: Mainly high moors but sometimes enclosed or afforested.

Land use: Mainly rough grazing but also woodland and occasional crops.

Soils: Peaty gleys,peaty podsols and peats but also other upland soils.

Vegetation:

LAND CLASS: TWENTY-THREE

Geography: N. England, C. and N. Scotland.

Land form: Ridges, scarps and corries leading to mountain Summits or rarely glaciated valleys

Topography: Extremely steep hillsides, sometimes less so, with the land at high altitudes.

Landscape: Open mountainous landscapes with wide vistas.

Land use: Limited open range grazing.

Soils: Peats, peaty podsols, podsols and brown rankers.

Vegetation: Mainly moorland types but also mountain grassland and peatland types.

LAND CLASS: TWENTY-FOUR

Geography: C. and W. Scotland.

Land form: Glaciated valley sides often reaching from base to rocky summits sometimes peaks emergent from peneplains.

Topography Precipitous and extremely steep slopes with land at high altitude.

Landscape: Rugged mountain scenery often rocky with fast flowing streams.

Land use: Limited open range grazing.

Soils: Brown rankers peats or peaty podsols, some peaty gleys.

Vegetation: Mainly peatland types but also mountain grassland and moorland.

LAND CLASS: TWENTY-FIVE

Geography: N.E. England, S. E., C. and N.E. Scotland.

Land form: Alluvial flood plains and morraines of glacial origin.

Topography: Virtually flat or gently rolling land mainly at low altitudes.

Landscape: Intensively farmed lowlands with fences and scattered farmhouses.

Land use: Mainly barley but with much good grassland.

Soils: Brown earths, gleys and gleyed brown earths.

Vegetation: Restricted to a few grassland types.

LAND CLASS: TWENTY-SIX

Geography: N.E. England, C. and E. Scotland.

Land form: Valley floors and coastal plains of glacial origin, sometimes with' emergent outcrops.

Topography: Undulating or smooth slopes mainly at low altitudes.

Landscape: Rather mixed lowland landscapes often affected by urban development.

Land use: Mainly good grassland but also much barley and pasture.

Soils: Brown earths and gleys.

Vegetation: Limited but mainly moorland types where present.

LAND CLASS: TWENTY-SEVEN

Geography: N. England, C., E. and N.E. Scotland.

Land form: Varied but mainly valley floors and bluffs occasionally with ridges and scarps.

Topography: Variable from mixtures of gentle and steep slopes to uniform moderate gradients mainly at medium low or low altitudes.

Landscape: Mainly well fenced lowlands, often mixed with woodland.

Land use: Arable, particularly barley but also much pasture and good grassland.

Soils: Brown earths and gleys.

Vegetation: Restricted but some grassland and moorland types.

LAND CLASS: TWENTY-EIGHT

Geography: N. England, S. and N.E. Scotland.

Land form: Heterogeneous from meandering riversides to peneplains or alluvial plains.

Topography: Mainly virtually flat but some gentle gradients at medium/low altitudes.

Landscape: Heterogeneous from enclosed farmed landscapes to open moorland.

Land use: Pasture or rough grazing predominate but some good grasslands also.

Soils: Variable but mainly gleys brown earths or peats.

Vegetation: Mainly peatland types where present but also grassland and moorland.

LAND CLASS: TWENTY-NINE

Geography: W. Scotland.

Land form: Indented coastlines with more cut platforms and raised beaches.

Topography: Uneven topography, usually with easy slopes but some steeper areas at low or medium/low altitudes.

Landscape: Complex scenery containing many contrasting elements.

Land use: Mainly open range grazing but also some crofting.

Soils: Mainly peats but also rankers and brown earths.

Vegetation: Mainly peatland and moorland types but also some bracken.

LAND CLASS: THIRTY

Geography: Extreme W. Scotland.

Land form: Mainly peneplains with meandering streams sometimes with low hills,

Topography: Variable from complex to almost flat at medium low extending to medium high altitudes.

Landscape: Open moorlands near to the sea with rocky outcrops and lochs.

Land use: Open range grazing and crofting.

Soils: Mainly peats with some peaty podsols.

Vegetation: Mainly peatland with some moorland types.

LAND CLASS: THIRTY-ONE

Geography: N. Scotland and Isles.

Landform: indented with some coastal plains backed by low hills.

Topography: Mainly broad gentle curved outlines and some steeper areas mainly at low/medium altitudes.

Landscape: Windswept, exposed coasts with the enclosed land divided into small fields.

Land use: Mainly rough grazing but some good grassland and pasture with crofting.

Soils: Brown earths peats and some podsols.

Vegetation: Mainly moorland but also some peatland and grassland types.

LAND CLASS: THIRTY-TWO

Geography: N.W. Scotland and Isles.

Land form: Peneplain surfaces or low ridges, sometimes coastal.

Topography: Variable from complex to even rounded slopes mainly at medium/low altitudes.

Landscape: Bleak moorlands often with scattered lochs and eroding peat hags.

Land use: Mainly open range grazing but some pasture.

Soils: Mainly peats but some rankers.

Vegetation: Predominantly peatland types but also some moorland.
