# Supplementary material for: Identification and predictability of soil quality indicators from conventional soil and vegetation classifications
Source: PLoS One. 2021 Oct 22;16(10):e0248665. doi: 10.1371/journal.pone.0248665 (PMC8535190; doi:10.1371/journal.pone.0248665)
Supplement: S2 Table — Number in brackets indicates the number of samples for that soil type. (DOCX) [file pone.0248665.s003.docx]

S2 Table: Shows conceptually comparable classification of the soils in the World reference base (WRB) Classification. Number in brackets indicates the number of samples for that soil type

| **Major soil type** | **Abbreviation** | **World Reference Base** |
| --- | --- | --- |
| Brown (96) | Browns | Cambisols, and some Luvisols, Acrisols |
| Lithomorphic (26) | Lithom | Leptosols and some Regosols |
| Ground/Surface water Gleys (38+68) | GWGs & SWGs | Gleysols, Planosols & some Fluvisols/Luvisols |
| Podzolic (34) | Podzol | Podzols |
| Peat (64) | Peat | Histosols |
| Pelosols (6) | Pelosol | Vertisols |
| N=304 |  |  |
