# Supplementary material for: Identification and predictability of soil quality indicators from conventional soil and vegetation classifications
Source: PLoS One. 2021 Oct 22;16(10):e0248665. doi: 10.1371/journal.pone.0248665 (PMC8535190; doi:10.1371/journal.pone.0248665)
Supplement: S3 Table — (DOCX) [file pone.0248665.s004.docx]

**S3 Table: The cross tabulation table of Aggregate Vegetation classes (AVCs) versus soil types.** The * shows AVCs that were under-sampled with respect to the soil type

|  | **Aggregate Veg class * Soil description Cross tabulation** | | | | | | | | |
| --- | --- | --- | --- | --- | --- | --- | --- | --- | --- |
|  |  | **Soil description** | | | | | | | **Total** |
|  |  | Browns | GWGs | Lithom | Peat | Pelosol | Podzol | SWGs |  |
| Crop and weeds | Count | 34 | 8 | 5 | 0* | 0* | 2* | 5* | 54 |
|  | Expected Count | 17 | 7 | 5 | 8 | 1 | 6 | 10 | 54 |
| Fertile grasslands | Count | 17 | 15 | 2* | 2* | 2* | 1* | 14 | 53 |
|  | Expected Count | 17 | 7 | 5 | 8 | 1 | 6 | 10 | 53 |
| Heath and bog | Count | 2* | 0* | 7 | 35 | 0* | 11 | 11* | 66 |
|  | Expected Count | 21 | 8 | 6 | 10 | 1 | 7 | 13 | 66 |
| Infertile grassland | Count | 24 | 10 | 7 | 1* | 2* | 6* | 12 | 62 |
|  | Expected Count | 20 | 8 | 5 | 9 | 1 | 7 | 12 | 62 |
| Lowland wooded | Count | 4 | 0* | 0* | 0* | 0 | 0* | 2 | 6 |
|  | Expected Count | 2 | 1 | 1 | 1 | 0 | 1 | 1 | 6 |
| Moorland grass mosaics | Count | 5* | 0* | 3 | 8 | 0* | 9 | 6 | 31 |
|  | Expected Count | 10 | 4 | 3 | 5 | 1 | 3 | 6 | 31 |
| Tall grass and herbs | Count | 6 | 3 | 0* | 0* | 0 | 0* | 2 | 11 |
|  | Expected Count | 3 | 1 | 1 | 2 | 0 | 1 | 2 | 11 |
| Upland wooded | Count | 4* | 2* | 2 | 0* | 2 | 5 | 6 | 21 |
|  | Expected Count | 7 | 3 | 2 | 3 | 0 | 2 | 4 | 21 |
| Total | Count | 96 | 38 | 26 | 46 | 6 | 34 | 58 | 304 |
